# Extra-Abdominal Desmoid-Type Fibromatosis Mimicking Myxofibrosarcoma

**DOI:** 10.5334/jbsr.2848

**Published:** 2022-09-21

**Authors:** Ye Rin Kim, Yu Sung Yoon, Hyerim Park

**Affiliations:** 1 From the Department of Radiology, Soonchunhyang University Bucheon Hospital, Soonchunhyang University College of Medicine, 170 Jomaru-ro, Bucheon 14584, Bucheon, Republic of Korea, KR; 2 From the Department of Radiology, Soonchunhyang University Cheonan Hospital, Soonchunhyang University College of Medicine, 31, Suncheonhyang 6-gil, Dongnam-gu, Cheonan-si, Chungcheongnam-do, Republic of Korea, KR

**Keywords:** paraspinal muscle, desmoid, fibromatosis, soft tissue tumor, magnetic resonance imaging

## Abstract

A 27-year-old woman was confirmed to have extraabdominal desmoid-type fibromatosis. Desmoid-type fibromatosis is a very rare connective tissue neoplasm with the extraabdominal type even more rare. It is most commonly found in proximal structures such as shoulders, neck, chest, and extremities. There are few case reports for desmoid-type fibromatosis located in paraspinal soft tissue. We report a case of desmoid-type fibromatosis mimicking a myxofibrosarcoma.

**Teaching Point:** Even if there are suggestive findings for malignant soft tissue tumor on radiologic evaluation, histological confirmation is necessary before surgical treatment.

## Introduction

Extraabdominal desmoid-type fibromatosis (DF) is a very rare condition that arises from the musculoaponeurotic structures [1,2]. Although pathologically benign and slowly growing, it can be locally infiltrative and proliferative tumors [1]. Because of their deep location, infiltrative growth pattern into adjacent subcutaneous tissue or muscle and its myxoid or fibrotic contents can mimic malignant soft tissue tumors. Therefore, it is difficult to differentiate based on these imaging findings.

The purpose of this case report is to inform that it is necessary to confirm the diagnosis by preoperative biopsy, even if there are imaging findings highly suggestive for malignancy.

## Case History

**Figure 1:**
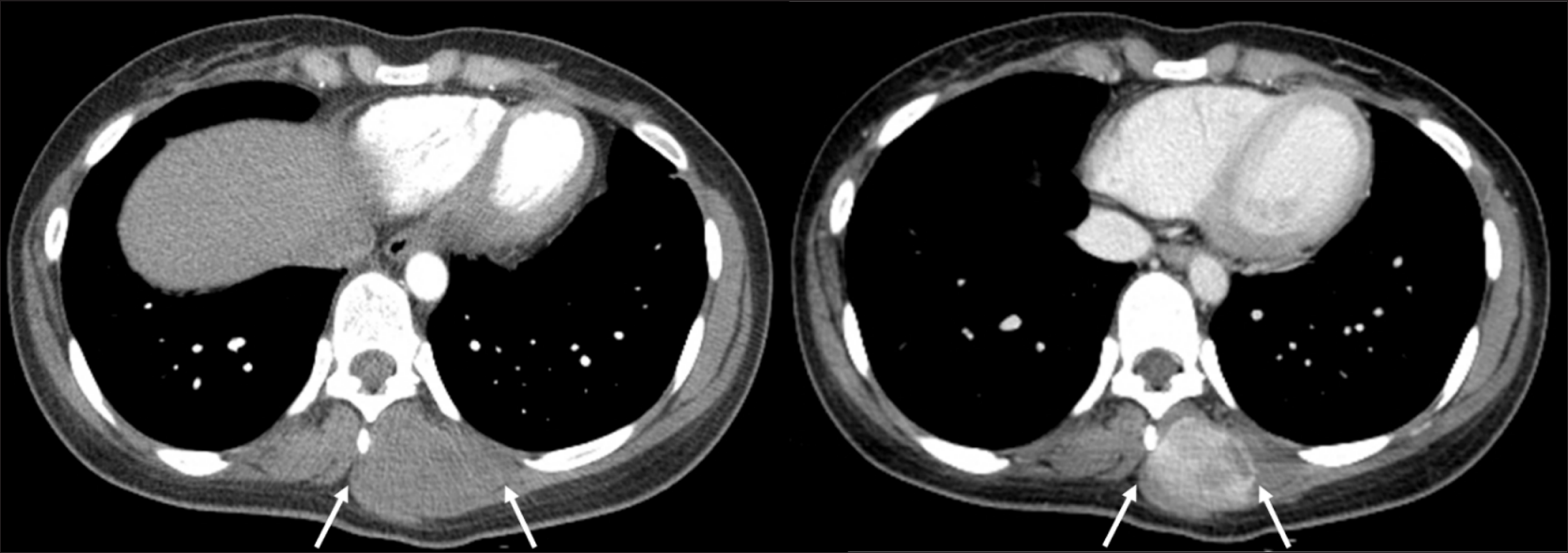
Arterial (*left*) and portal (*right*) phase on contrast-enhanced CT show a heterogeneously enhancing mass (*arrows*) in the left posterior paravertebral region of thoracic spine, centered on muscle group.

**Figure 2:**
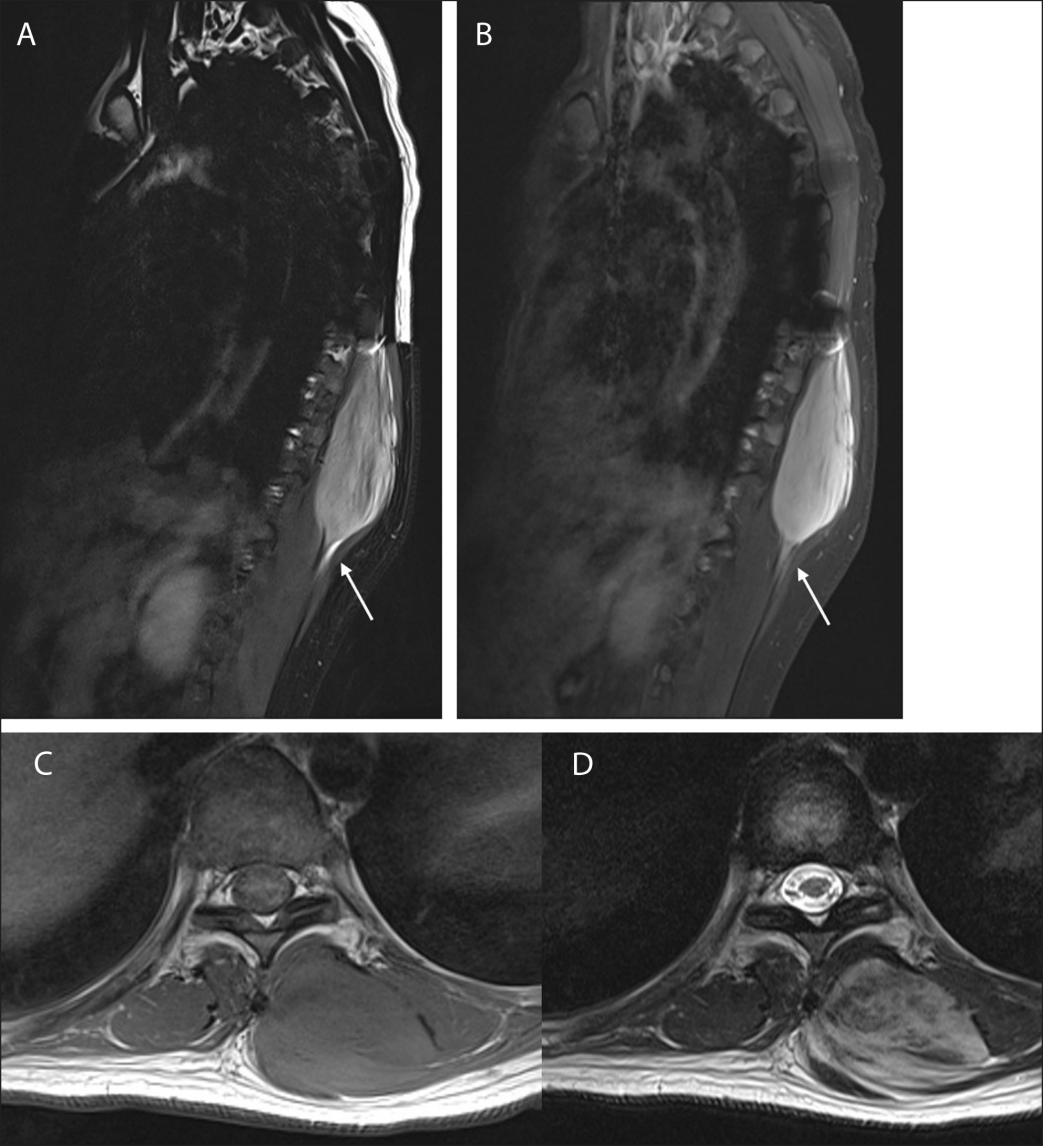
Contrast-enhanced thoracic spine MRI with sagittal **(A, B)** and axial **(C, D)** planes shows a well-defined 9.0 cm sized mass between the left erector spinae and lower part of trapezius muscle. This mass shows T1 isointensity (C), T2 hyperinensity (A, D), and intense contrast enhancement suggesting myxoid component (B), intralesional linear fibrotic component, and subfascial location with tail sign (*arrow*).

**Figure 3:**
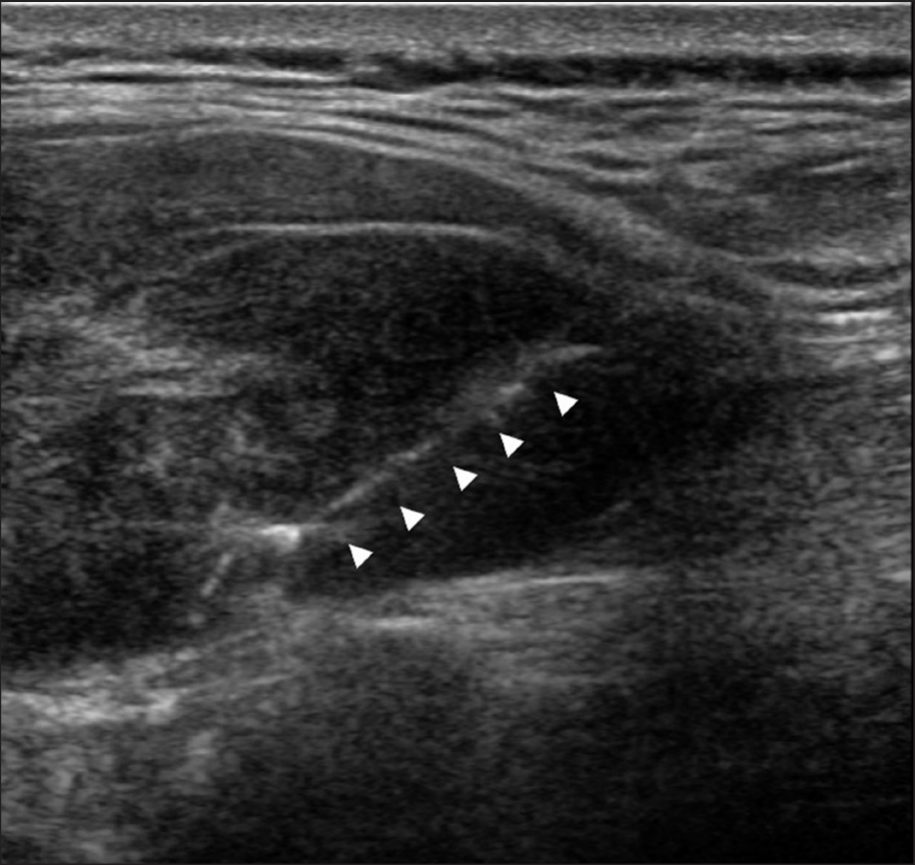
Ultrasonography shows heterogeneous hypoechoic mass with hyperechoic septum in the intermuscular space between erector spinae and trapezius muscles. Ultrasonography guided biopsy was performed with semi-automated co-axial needle. The specimen notch (*arrowheads*) located in solid enhancing portion based on MRI.

A 27-year-old woman presented with localized swelling and back pain for seven months. She had a history that included a traffic accident 21 months ago and had immediate T5 and T6 corpectomy with posterior fixation. Clinical examination revealed localized tenderness adjacent to the lower part of the operation scar on the back. The patient had no neurologic symptom or specific familial history. Contrast-enhanced computed tomography (CT) revealed a heterogeneously enhancing mass, located in the left posterior paraspinal soft tissue (Figure 1). Magnetic resonance imaging (MRI) showed a well-defined 9.0cm mass between the left erector spinae abutting the trapezius muscle (Figure 2). This mass showed T1 isointensity (Figure 2C), T2 hyperintensity (Figure 2A and 2D), intense contrast enhancement (Figure 2B), inner linear fibrotic component, and deep intermuscular location with tail sign. These imaging findings were consistent with the preprocedural working diagnosis of myxofibrosarcoma. Ultrasonography guided biopsy (Figure 3) was performed and pathologically confirmed extraabdominal DF.

## Comment

DF is a locally aggressive fibroblast and myofibroblast neoplasm, usually found in the deep intermuscular spaces of soft tissue. In addition, DF shows an invasive growth pattern and tends to have considerable rate of local recurrence after treatment [2]. Patients often report symptoms like a painless palpable mass in the lower extremity (e.g., thigh, foot, or ankle), upper extremity (e.g., shoulder or hand), or the head and neck [3]. DF occurs more frequently in females than males and between puberty and 40-year-old. Patients under the age of 30 years have a higher recurrence rate than those over their thirties. Factors associated with disease progression include changes in systemic hormones, prior trauma, surgical history, and pregnancy [1].

MRI is the most reliable technique for diagnosing DF which shows iso- to mildly hyperintense relative to muscle on T1-weighted images, and iso- to hyperintense relative to muscle on T2-weighted images with inner band-like areas showing low signal intensity (SI). However, because of the heterogeneity of the lesion, malignancy cannot be excluded on imaging. In our case, the large sized mass located in subfascial area of back muscles with T2 bright high SI and intense contrast enhancement suggested a myxoid component. Furthermore, the fibrotic band and peripheral tail sign, which are well-recognized prognostic factors of soft tissue sarcoma [4], were also shown. All these findings on MRI prior to biopsies raised suspicion of a malignant tumor such as myxofibrosarcoma. Therefore, biopsy and histopathologic confirmation had to be performed for appropriate therapeutic management.

For treatment of extraabdominal DF, surgical excision and/or radiation therapy can be considered. Hormonal therapy, such as tamoxifen or progesterone, or chemotherapy also may be considered for not easily resectable lesions [1]. Nevertheless, the local recurrence rate of extraabdominal DF is reported up to 77% [1,5,6]. Some studies report that this high local recurrence rate after surgical excision makes the ultimate outcome similar to observation or conservative treatment [6,7].

Although clinical and radiological evaluations suggest malignancy, histological confirmation before active treatment is essential.

## References

[1] McDonald ES, Yi ES, Wenger DE. Best cases from the AFIP: Extraabdominal desmoid-type fibromatosis. Radiographics. 2008; 28: 901–906. DOI: 10.1148/rg.283075169 18480491

[2] Ghanem M, Heinisch A, Heyde CE, Freiherr von Salis-Soglio G. Diagnosis and treatment of extraabdominal desmoid fibromatosis. GMS Interdiscip Plast Reconstr Surg DGPW. 2014 Feb 24; 3: Doc01. DOI: 10.3205/iprs000042 26504712 PMC4582506

[3] Kransdorf MJ. Benign soft-tissue tumors in a large referral population: Distribution of specific diagnoses by age, sex, and location. AJR Am J Roentgenol. 1995; 164: 395–402. DOI: 10.2214/ajr.164.2.7839977 7839977

[4] Spinnato P, Clinca R. MRI tail sign in soft-tissue sarcoma. Radiology. 2021; 299: 276. DOI: 10.1148/radiol.2021203877 33724067

[5] Teixeira LE, Arantes EC, Villela RF, Soares CB, Costa RB, Andrade MA. Extra-abdominal desmoid tumor: Local recurrence and treatment options. Acta Ortop Bras. 2016; 24: 147–150. DOI: 10.1590/1413-785220162403142182 27217816 PMC4863863

[6] Cuomo P, Scoccianti G, Schiavo A, et al. Extra-abdominal desmoid tumor fibromatosis: A multicenter EMSOS study. BMC Cancer. 2021; 21: 437. DOI: 10.1186/s12885-021-08189-6 33879110 PMC8059004

[7] Chew C, Reid R, O’Dwyer PJ. Evaluation of the long-term outcome of patients with extremity desmoids. Eur J Surg Oncol. 2004; 30: 428–432. DOI: 10.1016/j.ejso.2004.01.010 15063897

